# Antecedents and near-term consequences for interdisciplinary dissertators

**DOI:** 10.1007/s11192-017-2317-y

**Published:** 2017-03-27

**Authors:** Kevin M. Kniffin, Andrew S. Hanks

**Affiliations:** 1 Dyson School of Applied Economics and Management, S. C. Johnson College of Business, Cornell University, Warren Hall 111, Ithaca, NY 14853 USA; 2 Department of Human Sciences, The Ohio State University, Columbus, OH USA

**Keywords:** Interdisciplinary research, Wages, Risk, Immigrants

## Abstract

Given the complexity of questions studied by academicians, institutions are increasingly encouraging interdisciplinary research to tackle these problems; however, neither the individual-level pathways leading to the pursuit of interdisciplinary research nor the resulting market outcomes have been closely examined. In this study, we focus attention on the individuals who complete interdisciplinary dissertations to ask “who are they and how do they fare after earning the PhD?” Since interdisciplinary research is known to be relatively risky among academics, we examine demographic variables that are known to be associated in other contexts with risk-taking before considering whether interdisciplinarians’ outcomes are different upon graduating. First among our three main findings, students whose fathers earned a college degree demonstrated a 1.3% higher probability of pursuing interdisciplinary research. Second, the probability that non-citizens pursue interdisciplinary dissertation work is 4.6% higher when compared with US citizens. Third, individuals who complete an interdisciplinary dissertation tend to earn approximately 2% less in the year after graduation; however, mediation analyses show that the decision to become a postdoctoral researcher accounts for the apparent salary penalty. Our findings shed light on the antecedents and near-term consequences for individuals who complete interdisciplinary dissertations and contribute to broader policy debates concerning supports for academic career paths.

## Introduction

The products of interdisciplinary research have been relatively well-studied; however, comparable attention has not yet been paid to understanding the producers. Bibliometric research, for example, has typically focused on the relative impact of articles (e.g., Wang et al. 2015) and journals (Rafols et al. 2012) that integrate ideas from multiple disciplines without focusing attention on the consequences for authors of the respective products. As a complement to prior research, the novel focus of our interests centers on the individual-level question of “Who completes interdisciplinary dissertations and how do they fare after earning the PhD?”

A common starting point that our approach shares with prior research focused on bibliometric studies is that there is relatively greater risk associated with conducting interdisciplinary research when compared with more traditional discipline-focused pursuits (e.g., Van Noorden 2015). Against that backdrop, we examine demographic variables that are known to be associated in other contexts with risk-taking before considering whether interdisciplinarians’ salaries are different upon graduating. Among the benefits gained through our focus on individual-level interests, it is clear from Golde and Dore’s (2001) study of doctoral students’ career expectations that more empirical, systematic studies of typical career outcomes are needed. For example, while Golde and Dore (2001) focus on important career questions involving the probability of earning a tenure-track faculty position, there is clearly value to be gained by understanding how intrinsic market variables such as salary might be influenced by whether or not someone completes an interdisciplinary dissertation.

Beyond offering information for career-level decision-making, our interests to better understand the antecedents and near-term consequences for interdisciplinary dissertators has several additional justifications and benefits. First, in light of interests of colleges and universities to employ diverse workforces (e.g., Oldfield 2008), it is valuable to know whether people are disproportionately likely to pursue interdisciplinary postgraduate research as a function of demographic variables such as gender, citizenship status, and parental backgrounds. Second, against the backdrop of claims that globally important problems require more interdisciplinary integration (e.g., Schmidt et al. 2012), greater knowledge of any factors that currently appear to encourage people to work across disciplinary lines would be valuable. Finally, institution-level interests often encourage interdisciplinary pursuits on the basis of ideals just as academic analyses often highlight the synergies that can be gained by cross-fertilizing ideas across disciplinary boundaries (e.g., Bozeman 2013). Yet economic consequences such as income risk are typically either not understood or, at least, not closely considered. To partially fill this gap, our study examines whether individuals who pursue this path tend to face better or worse outcomes immediately after earning the PhD.

### Literature review and hypotheses

Academia is most commonly and directly regulated at the level of discipline-based departments through hiring and promotion. For example, Abbott (2001) argues that “as long as disciplinary academics act as the primary hiring agents for universities, they perpetuate the disciplinary system” (p. 126) and that “absent any radical change in the process of academic hiring, the current social structure of disciplines will endlessly recreate itself” (p. 127). Less directly but still importantly, hiring and promotion decisions at the department level are commonly and, in many cases, strongly influenced by decisions made by discipline-based journals and granting agencies to publish manuscripts and fund research proposals. With respect to these goals, Rhoten and Pfirman (2007, p. 68) stress the fact that “interdisciplinary papers are harder to review” since they are typically judged by people from a variety of disciplines who often have conflicting measures of quality. Similarly, notwithstanding administrative initiatives to provide interdisciplinary programs with hiring and promotion decisions (e.g., Ehrenberg 2004), Oberg (2009, p. 408) elaborates that interdisciplinary research is often assessed by reviewers according to discipline-specific biases in favor of certain methods (e.g., large-scale quantitative analysis) over others (e.g., case studies). In other words, people who specialize intensively in a discipline gain knowledge that is “largely tacit, situated, and experiential” (Kellogg et al. 2006, p. 24) that implicitly presents challenges and risks for would-be spanners. In a more explicit framework for evaluation, Rafols et al. (2012) add to the challenges of conducting interdisciplinary research by highlighting how journals that feature “mono-disciplinary research” tend to be more highly prized and valued than outlets that publish more intellectually diverse work.

The relative risk for individuals to conduct interdisciplinary research is reflected by Rhoten and Parker’s (2004) findings that “graduate students and full professors were indeed overrepresented” in their study of interdisciplinary programs when contrasted with the proportions of non-tenure-track faculty, postdoctoral researchers, and faculty at the assistant and associate ranks. Reasonable interpretations of this pattern recognize that graduate students have not yet committed as much time to any specific discipline and may be unaware of the potential labor market consequences. Full-rank professors, though, “have accumulated greater professional freedom and more social resources” (Rhoten and Parker 2004, p. 2046) and, consequently, are more able—at that point in their careers—to incur the risks associated with interdisciplinary work (Inkson et al. 2012). In fact, Sabharwal and Hu (2013) find that the productivity of full-rank professors appears to benefit disproportionately and uniquely with via participation in university-wide, department-spanning research centers. As for individuals who have made a significant investment of energy through completion of a doctoral program, the traditional discipline-based reward system would seem to explain why those most likely seeking promotion (e.g., to Associate or Full professor) tend to avoid interdisciplinary research.

Beyond Rhoten and Parker’s (2004) systematic study based upon career stage and the findings produced by Sabharwal and Hu (2013), there has been little attention focused on the demographic profile of people who conduct interdisciplinary postgraduate research; instead, as noted in our Introduction, bibliometric studies focused on products is much more common (e.g., Blackburn 1990; Pieters and Baumgartner 2002). In one exception, Falkenheim (2011) tabulates which specific universities tend to graduate the highest number of people who report interdisciplinary research activity. In a more substantive and sweeping exception, Rhoten and Pfirman (2007) test the hypothesis that women might participate disproportionately in interdisciplinary research because of a position that some have advanced that women are more inclined to think holistically, across disciplinary boundaries. While they report mixed results for their “women are more holistic” hypothesis, they are also clear about their main interest to draw more attention to the question of “who” pursues interdisciplinary postgraduate research.

Consistent with our motivation to understand the antecedents and consequences of decisions to conduct interdisciplinary postgraduate research, there are important ethical concerns that are relevant for programs that encourage people to produce relatively risky research. For example, while Rhoten and Pfirman (2007) focus on gender, their point can be applied much more broadly when they write that “using interdisciplinarity to attract women, as well as other underrepresented minority groups into science, is only practical and ethical if it leads to stable and secure pathways through scientific and academic careers” (p. 72). The relevance of this concern is illustrated clearly through the National Science Foundation’s (NSF) Integrative Graduate Education and Research Traineeship (IGERT) programs (e.g., Borrego and Newswander 2010; Moslemi et al. 2009; Schmidt et al. 2012) that are specifically geared to training graduate students to span academic boundaries. On the one hand, it makes sense for solution-driven projects to proceed without the burden of disciplinary biases on the grounds that new problems require new “disciplines.” On the other hand, though, there has been remarkably little investigation to date with respect to the individual-level outcomes that tend to obtain for graduate students who do engage in interdisciplinary studies.

The potential conflict of institution- and individual-level interests anticipated by Rhoten and Pfirman (2007) is best viewed as an extension of the more basic conflict of interest that people have debated with respect to recruiting individuals for any graduate program. For example, as Baird (1991) discovered, the number of graduate students in a department accounts significantly for the number of publications produced by a department’s faculty (e.g., in collaboration with graduate student researchers) even though “the publications rate of departments has little to do with educational outcomes for students” (p. 316). Against that backdrop, departmental efforts to recruit students for interdisciplinary research may reflect the department’s goal of broader recognition while not necessarily preparing graduate students for the associated risks.

Independent of one’s views on the implications of Baird’s findings, students agreeing to pursue interdisciplinary research may not fully consider, or even understand, the risks involved in such doctoral programs. Students may be “naïvely optimistic” (Golde and Dore 2001) about their postgraduate employment outcomes just as overly optimistic personalities may place disproportionate weight on positive outcomes (Weinstein 1980, 1989). Regardless of the reason, one of the motivations for our analyses is to generate knowledge concerning the typical pathways taken by individuals pursuing interdisciplinary postgraduate research. Individuals evaluating the benefits and risks associated with these programs will profit from a more systematic analysis of the antecedents and consequences for people enrolled in these programs.

In a risk-return framework, it makes sense that individuals postpone employment for graduate studies since there is a premium associated with the additional schooling (Autor et al. 2008). Yet, given the academic uncertainties related to interdisciplinary work, as well as the difficulty that interdisciplinary PhD candidates encounter in completing their studies (Newswander and Borrego 2009), it is an open question to consider whether interdisciplinary work is rewarded. Specifically, previous research has not quantified the rewards or risks associated with interdisciplinary dissertation research nor has the related subject of interdisciplinarians’ individual backgrounds been examined.

The three specific aspects that we address in the present article involve (1) the degree to which a person’s family background is associated with completing an interdisciplinary dissertation (among US citizens), (2) the degree to which citizenship, more broadly, is associated with completion of an interdisciplinary dissertation, and, (3) the relative rewards or risks associated with conducting interdisciplinary postgraduate research. Our approach presumes that understanding how doctoral recipients are distributed across demographic dimensions will supply university administrators and policy makers with information for developing relevant curricula and programs to produce successful PhD earners.

With respect to understanding the motivation of interdisciplinary degree seekers, the conventional view is that risk-taking behavior is a relative luxury. Investors, for example, commonly specify that any money invested in risky speculative stocks should be money that can be lost without great trouble (i.e., a category of money that most would consider to be a luxury). When applied to the questions that we are examining, the prediction is that people who belong to relatively privileged social groups will be more likely to pursue relatively risky interdisciplinary postgraduate research.

Focusing on ways in which a student’s socioeconomic background might influence their selection of undergraduate majors, it is notable that students whose parents did not earn a college degree tend to disproportionately pursue “vocational” degrees (e.g., in business, education, and engineering) while students with at least one parent who earned a college degree tend to pursue the relatively riskier “arts and sciences” (Goyette and Mullen 2006; Mullen et al. 2003; Wolniak et al. 2008). The same variable—whether or not someone is a first-generation college student—also appears to account for differences with respect to other aspects of academic career paths (e.g., Kniffin 2007), including the pursuit of risky graduate degrees. Drawing on data from the 2002 *Survey of Earned Doctorates* conducted by the National Science Foundation (NSF), Hoffer et al. (2003, p. 36) report: “Compared to doctorate recipients with higher levels of parental education, the first-generation graduates were over-represented in education … and underrepresented in humanities and, to a lesser extent, social sciences and physical sciences.” In a separate survey of more than 9000 doctoral students from 21 research universities in the US, Nettles and Millett (2006) find a similar pattern whereby the percentage of graduate students with at least one parent with a doctoral or professional degree ranges from 16% in the least-risky field of education to 24, 26, 27, and 34% for students, respectively, enrolled in engineering, social science, science, and humanities doctoral programs. Projecting from those patterns identified by prior research concerning the selection of undergraduate and graduate majors as a function of parental education levels—whereby riskier degree programs tend to be selected by students whose parents have more formal educations (Goyette and Mullen 2006; Mullen et al. 2003; Wolniak et al. 2008), we hypothesize that students—among US citizens—who complete interdisciplinary dissertations will tend to have parents who have more formal education than other recent dissertators.

#### **Hypothesis 1**

People whose parents have relatively more formal education will be more likely to pursue interdisciplinary postgraduate research.

Comparing US citizens with non-citizens across industries, immigrants to the US tend to disproportionately pursue entrepreneurial goals (e.g., Bogan and Darity 2008; FPI 2012). Fixed into the narrative of the US as a “bastion of opportunity,” the tradition of immigrants founding companies has a long history and cuts across industries (e.g., Ndofor and Priem 2011). While much of the popular focus on immigrants opening their own businesses has focused on retail establishments, there is ample evidence that immigrants also contribute significantly—and disproportionately—to innovations in a wide range of skilled professions. Hunt and Gauthier-Loiselle (2010), for example, report that one percent increases in the number of skilled immigrants in the US tend to yield approximately 15% increases in patents per capita. Immigrants are not directly responsible for the full effect; instead, interestingly, their direct contribution to increased patent production appears to have positive spillover effects that help spur more patent claims by non-immigrants.

Within the industry of academics, the integration and application of research concerning immigrants in other industries lends itself to the prediction that non-citizens in the US will be more likely to pursue interdisciplinary postgraduate research. The importance of this relationship is clear in light of the significant increase in non-citizens earning research doctorates in the US (Chang and Milan 2012). Among doctoral recipients in the US in the natural sciences and engineering, for example, Stephan (2012) reports an increase in non-citizens from 20% in 1966 to approximately 46% in 2010. Consistent with this trend, Mervis (2008) provocatively recognized in *Science* that the “Top Ph.D. Feeder Schools [to the US] Are Now Chinese.” In the current research, our focus is not on students’ specific country of origin or choice of discipline (Stephan 2012). Instead, we consider the full array of doctoral recipients rather than limiting our interests on those in the sciences and engineering (Grogger and Hanson 2013)—or, say, management (Seibert et al. 2017)—and we examine the degree to which immigrants pursuing the PhD exhibit the risk-taking entrepreneurial traits of immigrants in other industries. Evidence from our analysis supports the notion that in academia, immigrants to the US still exhibit an entrepreneurial spirit.

More qualitatively with respect to the individual-level decisions that are made, there is a notable selection bias whereby individuals who emigrate to the US tend to have a different degree of ambition—focused on their family’s future—and, arguably, a different kind of imagination whereby they leave their native country—most typically, from a different continent—to pursue years of graduate research in the US (Kannankutty and Burrelli 2007). This general characterization of immigrant graduate students fits with the proposition that non-citizen graduate researchers are more likely to take risks to address important gaps in knowledge. Given a reasonable assumption that gaps in knowledge are more likely to occur between or across disciplinary boundaries, we expect that non-citizen graduate students will be disproportionately represented among those pursuing interdisciplinary postgraduate research.

#### **Hypothesis 2**

People who immigrate to the United States will be more likely to conduct interdisciplinary postgraduate research.

Focusing on the relative rewards or risks, we hypothesize that there are near-term risks associated with interdisciplinary dissertation work. In general, we expect that there is a potential conflict between the organizational benefits that interdisciplinary dissertators can generate (e.g., problem-specific solutions) and their own individual outcomes. This expectation fits with Hackett and Rhoten’s (2009) findings that suggest that graduate students typically learn to shy away from interdisciplinary work before earning the PhD since “disciplines offer reliable recipes for the production of certified knowledge” (2009, p. 424).

While we expect variation across career stages with respect to the risks and rewards for interdisciplinary research, our initial investigation focuses on the near-term post-doctorate experience for which we expect the conservative nature of the job market tends to apply a *de facto* penalty. One reason we expect negative outcomes in the near-term is that the relative lack of signals that are available for the full population of newly minted PhD-level researchers is further clouded with “confusion and ambiguity” (Durand and Paolella 2013) when someone’s work cuts across traditional boundaries and categories. Our hypothesis is also presumed—but untested—by the conventional wisdom that after an assistant professor receives tenure, then—but not before—they are positioned to take greater intellectual risks (Leahey et al. 2010).

#### **Hypothesis 3**

People who conduct interdisciplinary postgraduate research will tend to have relatively inferior near-term career outcomes.

## Data and methods

### Data

The annual Survey of Earned Doctorates (SED) conducted by the National Science Foundation’s (NSF) National Center for Science and Engineering Statistics (NCSES) presents the ideal dataset for testing our models. To focus on the most recent year of available data, we utilized responses from the 2010 edition of the Survey, which was administered to everyone earning a research doctorate in the US between July 1, 2009 and June 30, 2010. Fiegener (2011) reports that the 2010 Survey gained responses from 92.9% of the 48,609 people who earned the doctorate that year in the US.

For people who do not complete the full survey, the SED records limited information based upon “administrative lists of the university, such as commencement programs and graduation lists.” For example, Fiegener (2011) reports that gender is recorded for 99.97% of respondents and citizenship is known for 94.0% of the population of doctorate graduates from 2010. With respect to various kinds of doctoral degrees, the 2010 SED primarily concerns people who earned the Doctor of Philosophy (PhD) (95.8%) and Doctor of Education (EdD) (3.1%) and does not involve people with “professional doctorates” in law, medicine, or dentistry.

For the purposes of this study, we mainly focus on PhD earners who declared US citizenship partly because measures of socioeconomic background as well as culture-specific attitudes to higher education are variable across countries (e.g., Daouli et al. 2010; Sen and Clemente 2010). Our approach omits significant heterogeneity, which can greatly affect standard errors for the point estimates, for our primary analyses. We do, however, examine the impact of US citizenship on the decision to pursue an interdisciplinary degree and are able to compare the 29,568 respondents who are US citizens (65.6%) with the 15,516 respondents in our sample who are immigrants (34.4%).

### Variables

#### Interdisciplinary postgraduate research

Notwithstanding broader debates about the definition of interdisciplinarity (e.g., Huutoniemi et al. 2010), we followed previous researchers (Falkenheim 2011; Millar 2013; Millar and Dillman 2010a, b) who categorized respondents to the SED who indicated a secondary field for their degree as people who pursued interdisciplinary postgraduate research. More specifically, the 2010 Survey prompted respondents with the following text: “If your dissertation was interdisciplinary, list the name and number of your secondary field.” We also control for the primary dissertation field since individuals in some fields are disproportionately likely to pursue interdisciplinary work.

#### Demographic variables

Among the background variables that are measured by the Survey, our analysis utilizes measures of Age (or Year of Birth), Gender, Ethnicity, Citizenship, and parental education. For parental education, respondents are asked to provide one of eight options for each parent, indicating whether an individual’s mother and father received anywhere from no education to an advanced degree. Based on previous research described above, we collapsed the range to focus on potential differences as a function of whether a person’s mom (*MotherEdu*) or dad (*FatherEdu*) earned a college degree. Throughout our analyses, we adopt the same category labels (e.g., for ethnic categories) as the NSF used in its Survey instrument.

We also utilize the Carnegie classification system to control for university research intensity given evidence that (1) the pedigree of a doctoral student’s institution is an important predictor for hiring in the year after graduation and (2) institutional pedigree is strongly related to research intensity (Hilmer and Hilmer 2012). While this classification system does not include institution-specific rankings per se, it identifies PhD granting institutions as having very high, high, or moderate research activity. These three types of PhD granting institutions represent nearly 95% of the universities in the sample. This classification system also identifies smaller PhD granting institutions that might have minimal or non-existent research activity. For our analysis, we create indicator variables for each of the classifications mentioned above. The variables *CarnegieClass*2*, CarnegieClass*3, and *CarnegieClass*4 are included in the regressions, with the lower classes (1–3) representing universities with the most research activity. The variable *CarnegieClass*1, representing universities with the highest research activity, is left out and serves as the reference category.

#### Near-term consequences

Drawing upon responses to questions about post-graduation plans, we utilized answers to the prompts: (1) “Do you intend to take a ‘postdoc’ position?” and (2) “What will be your basic annual salary for this principal job (in the next year)? Do not include bonuses or additional compensation for summertime teaching or research. If you are not salaried, please estimate your earned income.” While the question regarding postdocs provided two options (yes or no), respondents were invited to select one of 12 options to report their salary, ranging from “$30,000 or less” to “$110,000 or above” with an additional option to indicate that they “Don’t know” their salary for the year after earning the doctorate. Salary ranges spanned $5000 for the first 2 brackets and then $10,000 thereafter. To facilitate interpretation of regression coefficients, we used the means of the salary ranges as values for the dependent variable.

### Specifications

Ultimately, the near-term outcome of interest is how the employment market compensates the new PhD recipient. Yet as discussed above, the pathways that lead to this provide important information about the factors influencing salary. Upon entrance into a PhD program in a given field, an individual becomes subject to the market forces determining how PhD degrees from this field are compensated. Yet there is variation in the salaries offered to these individuals and our objective is to characterize some of that variation in terms of the research that individuals pursued. Our use of the interdisciplinary variable is to help capture some of the variation in PhD salaries after controlling for other background characteristics and primary discipline. Indeed, these very background characteristics as well as primary discipline are also important pathways leading to whether or not an individual will choose to pursue an interdisciplinary degree.

We also consider postdoctoral research positions—accepted by 43% of the entire sample—as another market outcome that is influenced in part by interdisciplinary research pursuits. Interestingly, however, some fields expect postdoctoral training prior to obtaining a position at a research university. Thus the field of study is clearly an important factor in determining whether or not an individual pursues and/or accepts a postdoctoral research position. In other fields, the decision to pursue postdoctoral research is not determined until the candidate is searching for a position. Thus to the degree that interdisciplinary research influences the propensity to pursue postdoctoral research, holding constant the primary disciplinary field, it is very possible that the pursuit of a postdoctoral research position mediates the direct effect of interdisciplinary research on salary. In other words, individuals who pursue interdisciplinary research make the decision before entering the job market. Once they are on the job market, the actual job they obtain, postdoctoral position or not, directly influences salary.

To study the pathways influencing interdisciplinary research, the effect of interdisciplinary research on the propensity to accept a postdoctoral research position, whether or not interdisciplinary research directly influences salary, and whether or not pursuing postdoctoral research mediates the direct effect of interdisciplinary research on salary, we will estimate the three equations specified below. We indicate that all analyses were done using Stata v.14, developed by StataCorp LP.

First, to understand how interdisciplinary degree seekers are distributed across demographic characteristics, we assume a linear relationship between the propensity to pursue an interdisciplinary degree, degree field, Carnegie classification indicator variables, and demographic characteristics. The linear model we use to test this relationship is given by

1 $$ \begin{aligned} IntDisc_{i}^{*} & = \alpha_{0} + \alpha_{1} FatherEdu + \alpha_{2} MotherEdu + \alpha_{3} Field_{1i} + \alpha_{4} Field_{2i} \\ & \quad + \alpha_{5} Field_{3i} + \alpha_{6} Field_{4i} + \alpha_{7} Field_{5i} + \alpha_{8} Field_{6i} + \alpha_{9} Field_{7i} + \alpha_{10} Field_{8i} + \alpha_{11} Field_{9i} \\ & \quad + \alpha_{12} Field_{10i} + \alpha_{13} CarnegieClass1 + \alpha_{14} CarnegieClass2 \\ & \quad + \alpha_{15} CarnegieClass3 + \alpha_{16} BirthYear + \alpha_{17} USCit_{i} + \alpha_{18} Gender_{i} + \varepsilon_{INTi} , \\ \end{aligned} $$

where *Salary*_*i*_ is the salary individual *i* will receive post graduation, *IntDisc*_i_^*^ is the propensity of individual *i* to pursue an interdisciplinary degree, *FatherEdu* is the individual’s paternal education level, *MotherEdu* is the individual’s maternal education level, *Field*_1–10_ denote the individual’s primary dissertation field within one of the main disciplinary categories, *CarnegieClass*2–4 categorizes universities by Carnegie classifications where level 1 (omitted) represents very high research activity, level 2 represents high research activity, and level 3 represents moderate research activity, and level 4 represents smaller universities or colleges, *BirthYr* is the individual’s year of birth, *Gender* is the individual’s gender, *White* is the individual’s ethnicity, and $\varepsilon_{PDi}$ is an independent and identically distributed random error term.

The variable *IntDisc*_i_^*^ is the propensity of individual *i* to pursue an interdisciplinary degree, and the remaining variables are as described in Eq. (1) and $\varepsilon_{INTi}$ represents the unobserved effects not captured by the independent variables, and is assumed to be independent and identically distributed. The subscript *INT* on the random error term $\varepsilon$ in Eq. (1) identifies the random error term in relation to the decision to pursue interdisciplinary research. The dependent variable, *IntDisc*_i_^*^ is unobserved so a binary variable is used and equals 1 when interdisciplinary research was specified, and 0 otherwise. A probit estimation procedure is used to estimate the vector *α* of unknown parameters.

Second, we estimate a mediation model to determine the degree to which postdoctoral research pursuits mediate the choice to engage in interdisciplinary research. The decision to write an interdisciplinary dissertation most likely occurs well before an individual decides to accept a postdoctoral position. Of course there are disciplines in which postdoctoral positions are a necessary step in a research career, but it is possible that individuals are not certain they want to continue on a research path until they approach graduation and observe the job market. Thus it is very plausible that individuals decide to conduct interdisciplinary research for their PhD. We expect that this decision increases the chances that individuals accept a postdoctoral research position immediately after graduation. In turn, the postdoctoral research position results in a lesser salary in the first year after receiving the PhD. In other words, we hypothesize that interdisciplinary research influences salary through the choice to take a postdoctoral research position.

In the regressions for the mediation models, we again control for individual background characteristics, primary discipline, as well as the Carnegie classification of the school. Thus, in the following specifications, we estimate the effect that interdisciplinary research and other demographic factors have on the decision to accept a postdoctoral position after receipt of the PhD. Then we estimate the degree to which accepting a postdoctoral research position mediates the impact of interdisciplinary research on salary. The estimation equations are given by

2 $$ \begin{aligned} PostDoc_{i}^{*} & = \beta_{0} + \beta_{1} IntDisc + \beta_{2} FatherEdu_{i} + \beta_{3} MotherEdu_{i} + \beta_{4} Field_{1i} + \beta_{5} Field_{2i} \\ & \quad + \beta_{6} Field_{3i} + \beta_{7} Field_{4i} + \beta_{8} Field_{5i} + \beta_{9} Field_{6i} + \beta_{10} Field_{7i} + \beta_{11} Field_{8i} \\ & \quad + \beta_{12} Field_{9i} + \beta_{13} Field_{10i} \\ & \quad + \beta_{14} CarnegieClass1 + \beta_{15} CarnegieClass2 + \beta_{16} CarnegieClass3 \\ & \quad + \beta_{17} BirthYr_{i} + \beta_{18} Gender_{i} + \beta_{19} White_{i} + \varepsilon_{PDi} , \\ \end{aligned} $$

where *PostDoc*_i_^*^ is the propensity of PhD candidate *i* to accept a postdoctoral position following graduation and the remaining variables are the same as those used in Eq. (1). Again, note that the subscript *PD* on the random error term, *ε*, specifies that these terms correspond specifically to Eq. (2) and its focus on predicting postdoctoral employment. Also, *PostDoc*_i_^*^ is not observed thus we use a binary variable that equals 1 when an individual selected a postdoctoral research position and 0 otherwise. Similar to Eq. (1), a probit estimation procedure is used to estimate the parameters in the model.

Finally, our expectation that interdisciplinary research pursuits affect the choice to accept a postdoctoral position leads to the following two equations in the mediation analysis. As an initial step (Eq. 3a), we estimate if there is a direct effect of interdisciplinary research on salary. Then we estimate the salary Eq. (3b) and include both the interdisciplinary research variable and the postdoctoral position variable. We also include the same set of covariates as in the previous two models.

3a $$ \begin{aligned} Salary_{i} & = \gamma_{0} + \gamma_{1} IntDisc_{i} + \gamma_{2} FatherEdu_{i} + \gamma_{3} MotherEdu_{i} \\ & \quad + \gamma_{4} Field_{1i} + \gamma_{5} Field_{2i} + \gamma_{6} Field_{3i} + \gamma_{7} Field_{4i} + \gamma_{8} Field_{5i} + \gamma_{9} Field_{6i} \\ & \quad + \gamma_{10} Field_{7i} + \gamma_{11} Field_{8i} + \gamma_{12} Field_{9i} + \gamma_{13} Field_{10i} + \gamma_{14} CarnegieClass2 \\ & \quad + \gamma_{15} CarnegieClass3 + \gamma_{16} CarnegieClass4 + \gamma_{17} BirthYr_{i} + \gamma_{18} Gender_{i} \\ & \quad + \gamma_{19} White_{i} + \varepsilon_{INCi} , \\ \end{aligned} $$

3b $$ \begin{aligned} Salary_{i} & = \delta_{0} + \delta_{1} IntDisc_{i} + \lambda_{1} Postdoc + \delta_{2} FatherEdu_{i} + \delta_{3} MotherEdu_{i} \\ & \quad + \delta_{4} Field_{1i} + \delta_{5} Field_{2i} + \delta_{6} Field_{3i} + \delta Field_{4i} + \delta_{8} Field_{5i} + \delta_{9} Field_{6i} \\ & \quad + \delta_{10} Field_{7i} + \delta_{11} Field_{8i} + \delta_{12} Field_{9i} + \delta_{13} Field_{10i} + \delta_{14} CarnegieClass2 \\ & \quad + \delta_{15} CarnegieClass3 + \delta_{16} CarnegieClass4 + \delta_{17} BirthYr_{i} + \delta_{18} Gender_{i} \\ & \quad + \delta_{19} White_{i} + \varepsilon_{INCi} , \\ \end{aligned} $$

The *INC* subscript on the random error term, *ε*, denotes that these variables correspond to the equation estimating the impact of factors on income or salary.

Since salary ranges are censored both above and below, standard linear regression techniques will generate inconsistent coefficient estimates and incorrect standard errors. To correct for this specification problem, a double-censored Tobit regression technique is used. This technique accounts for the probability mass that builds up at the censoring points as defined in the survey–$30,000 and $110,000 in this case–and generates appropriate estimates and standard errors.

## Results

As indicated in Table 1, a significant percentage of individuals who earn doctoral degrees engage in interdisciplinary research. In fact, among those whose primary field is in the Agricultural and Life Sciences, 44% of respondents reported their work as interdisciplinary. Surprisingly, since the disciplines would seem to be closely related, the second-lowest percentage of interdisciplinary dissertations (27%) was found among people in the Social Sciences.

**Table 1:** Percentage of doctorates awarded by discipline and interdisciplinary focus

Discipline	% All research doctorates	% Interdisciplinary
Agricultural and Life Sciences	2.3	44.5
Biological Sciences	17.6	41.1
Health Sciences	4.4	29.9
Engineering	16.0	32.8
Computer Sciences And Mathematics	7.0	22.7
Physical Sciences	10.9	29.3
Social Sciences	16.2	26.9
Humanities	10.6	37.7
Education	11.0	29.4
Business Management	2.8	31.2
Communications	1.4	39.8

Descriptive measures of the sample of US citizens are given in Table 2. In the sample, 30.2% of US citizens who earned research doctorates in 2010 chose to pursue interdisciplinary dissertation work, 52% were women, 82.6% were White or European American, more than half of their mothers and/or fathers had earned a college degree, and their average age was 36. Correlation coefficients for the variables of interest also indicate potential contributors to the decision to pursue interdisciplinary work and factors that may influence salary.

**Table 2:** Descriptive statistics and correlations among US citizens whose primary disciplines are classified in Table 1

Variable	Mean	SD	1	2	3	4	5	6	7	8	9	10	11	12	13
1. Interdisciplinary dissertation	30.1%	0.459													
2. Father w/college degree	59.7%	0.491	0.04*												
3. Mother w/college degree	52.8%	0.499	0.03*	0.54*											
4. Salary	$58,210	23.237	−0.04*	−0.05*	−0.06*										
5. Post Doc	38.4%	0.486	0.06*	0.07*	0.08*	−0.50*									
6. Birth Year	1974.22	8.600	−0.02*	0.22*	0.25*	−0.24*	0.28*								
7. Female	51.3%	0.500	0.01	−0.04*	0.02*	−0.13*	−0.02*	−0.09*							
8. Carnegie classification 1	71.8%	0.378	0.03*	0.15*	0.15*	−0.07*	0.12*	0.24*	−0.07*						
9. Carnegie classification 2	17.2%	0.223	−0.03*	−0.10*	−0.10*	0.04*	−0.10*	−0.15*	0.03*	−0.73*					
10. Carnegie classification 3	5.7%	0.233	−0.01*	−0.09*	−0.09*	0.11*	−0.09*	−0.20*	0.05*	−0.39*	−0.11*				
11. Carnegie classification 4	5.3%	0.223	0.00	−0.05*	−0.04*	−0.04*	0.03*	−0.02*	0.03*	−0.38*	−0.10*	−0.06*			
12. White	83.3%	0.373	−0.01*	0.10*	0.07*	−0.03*	−0.02*	0.03*	−0.05*	0.03*	0.01	−0.05	−0.02*		
13. Time to Degree (years)	7.88	1.907	0.02*	−0.13*	−0.14*	0.17*	−0.28*	−0.63*	0.07*	−0.15*	0.11*	0.13*	−0.01	−0.06*	
14. Industry	7.7%	0.266	−0.02*	0.02*	0.02	0.36*	−0.16*	0.02*	−0.09*	0.00	−0.01	0.02*	−0.01	−0.01	−0.04*

* *p* < 0.05

**Fig. 1:**
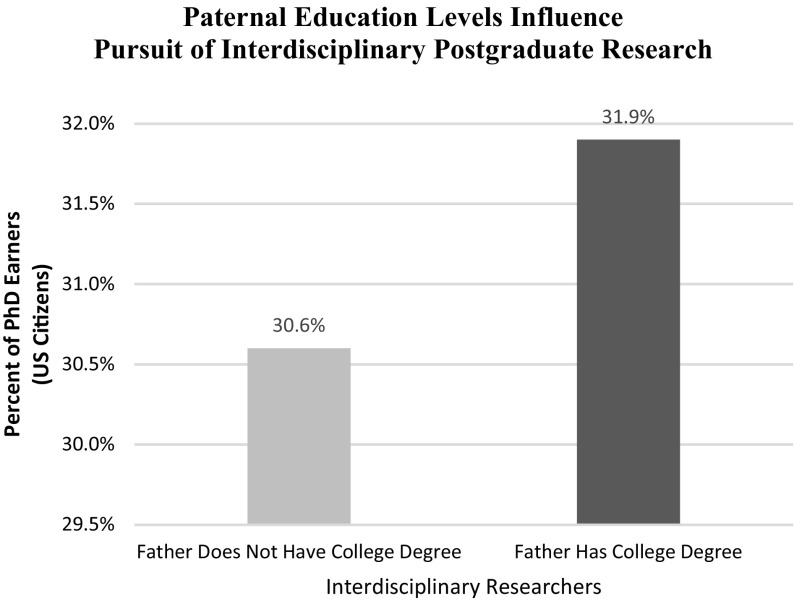
Paternal education levels influence pursuit of interdisciplinary postgraduate research

To understand how interdisciplinary degree seekers are distributed across socioeconomic and other dimensions, we estimated the parameters of Eq. (1). As indicated in Table 3, we find that parental education levels—specifically whether a student’s father earned a college degree—was weakly important. More specifically, when their father earned a college degree, the percentage of individuals who pursued an interdisciplinary dissertation project increased by 1.3 points as illustrated in Fig. 1. While it is interesting that paternal—and not maternal—education is important, the findings offer mixed support for Hypothesis 2 whereby people from families with more formal education may engage, with greater probability, the risk of interdisciplinary postgraduate research. Table 3 also indicates no significant influence for gender and, curiously, white doctoral students tend to significantly avoid interdisciplinary dissertation research. On the other hand, a greater percentage of individuals from the universities with the highest research activity tend to pursue interdisciplinary research.

**Table 3:** Impact of socioeconomic background on interdisciplinary research

Variable	Coefficient	Standard error	*Z*-statistic	*P* value
*Panel A Dependent variable: choose interdisciplinary degree*				
Father Education	0.04	0.02	1.87	0.06
Mother Education	0.00	0.02	−0.22	0.83
Biological Sciences	−0.04	0.06	−0.74	0.46
Health Sciences	−0.45	0.07	−6.93	0.00
Engineering	−0.29	0.06	−4.81	0.00
Computer Sciences and Mathematics	−0.53	0.07	−8.07	0.00
Physical Sciences	−0.40	0.06	−6.69	0.00
Social Sciences	−0.46	0.06	−8.05	0.00
Humanities	−0.16	0.06	−2.80	0.01
Education	−0.45	0.06	−7.72	0.00
Business Management	−0.31	0.08	−4.09	0.00
Communications	−0.12	0.08	−1.47	0.14
University w/High Research Activity	−0.06	0.02	−2.71	0.01
University w/Moderate Research Activity	−0.03	0.04	−0.84	0.40
PhD Granting College or University	−0.02	0.04	−0.53	0.60
Birth Year	−0.01	0.00	−8.37	0.00
Female	0.03	0.02	1.80	0.07
White	−0.08	0.02	−3.78	0.00
Constant	17.76	2.14	8.31	0.00
*Panel B Marginal effects of interdisciplinary degree*				
Father: No College Education	30.6%	0.005	61.8	0.00
Father: College Education	31.9%	0.004	84.0	0.00

Method: cross-section probit specification

Dependent variable: (0/1) completion of interdisciplinary dissertation

To understand the extent to which interdisciplinary dissertators might face opportunity costs that would be demonstrated by their “Time to Degree,” Table 2 includes the relevant values and shows a slight but significant positive correlation (*r* = .02, *p* < 0.01) between (a) completing an interdisciplinary dissertation and (b) how long it takes to complete a doctoral program. A closer look at Row 13 in Table 2, though, shows that Time to Degree is also significantly correlated with an array of other variables. For example, age (or Birth Year) is substantially and inversely related with Time to Degree (*r* = −.63, *p* < 0.01) perhaps reflecting older students who are concurrently employed or semi-retired. Regression analyses that focus on the relevance of completing an interdisciplinary dissertation for Time to Degree and control for the set of disciplinary and demographic variables considered in our other analyses show that there is not a meaningful relationship between completing an interdisciplinary dissertation and the Time to Degree.

**Fig. 2:**
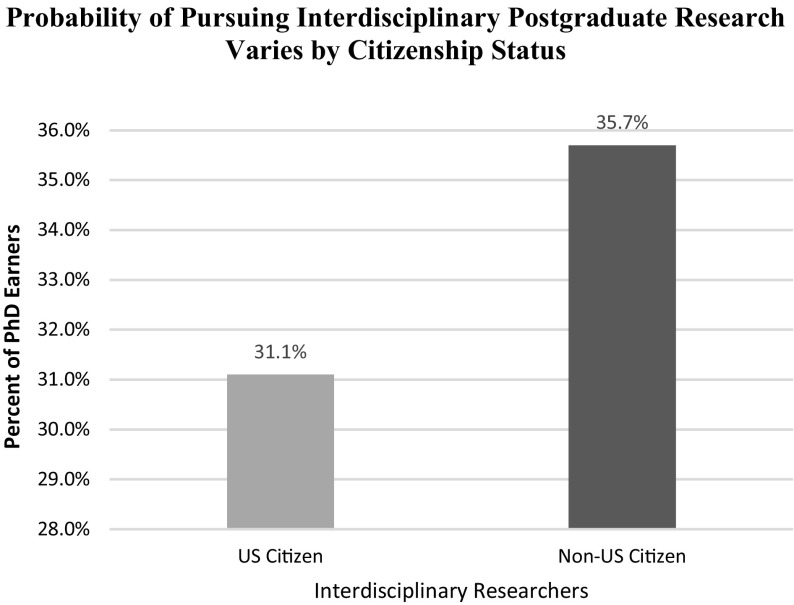
Probability of pursuing interdisciplinary postgraduate research varies by citizenship status

In addition, results in Table 4 (also Fig. 2) support Hypothesis 2 and show that the probability that immigrants to the United States—non-citizens, more precisely—choose to conduct interdisciplinary research for their doctoral work increases by 4.6% points. Notably, the results for the model used in this analysis indicate that gender does not contribute significantly to predicting the pursuit of interdisciplinary dissertation when citizens and non-citizens are compared. In addition, the university’s research activity, as characterized by the Carnegie classifications, had no impact when examining the full sample of individuals receiving a PhD.

**Table 4:** Impact of citizenship status on interdisciplinary research

Variable	Coefficient	Standard error	*Z*-statistic	*P* value
*Panel A Dependent variable: choose interdisciplinary degree*				
Biological Sciences	−0.05	0.04	−1.05	0.29
Health Sciences	−0.40	0.05	−7.86	0.00
Engineering	−0.30	0.04	−6.88	0.00
Computer Sciences and Mathematics	−0.59	0.05	−12.28	0.00
Physical Sciences	−0.38	0.04	−8.42	0.00
Social Sciences	−0.45	0.04	−10.35	0.00
Humanities	−0.16	0.04	−3.48	0.00
Education	−0.42	0.05	−9.13	0.00
Business Management	−0.37	0.06	−6.63	0.00
Communications	−0.12	0.07	−1.74	0.08
University w/High Research Activity	−0.03	0.02	−1.48	0.14
University w/Moderate Research Activity	−0.05	0.03	−1.64	0.10
PhD Granting College or University	−0.00	0.03	−0.06	0.95
Birth Year	−0.01	0.00	−9.26	0.00
US Citizen	−0.13	0.01	−9.23	0.00
Female	0.00	0.01	0.16	0.88
Constant	17.39	1.89	9.22	0.00

	Predicted Probabilities (%)	Standard error	*Z*-statistic	*P* value
*Panel B Marginal effect of US citizenship*				
Non-US Citizen	35.7	0.004	86.63	0.00
US Citizen	31.1	0.003	110.49	0.00

Method: cross-section probit specification

Dependent variable: (0/1) completion of interdisciplinary dissertation

**Fig. 3:**
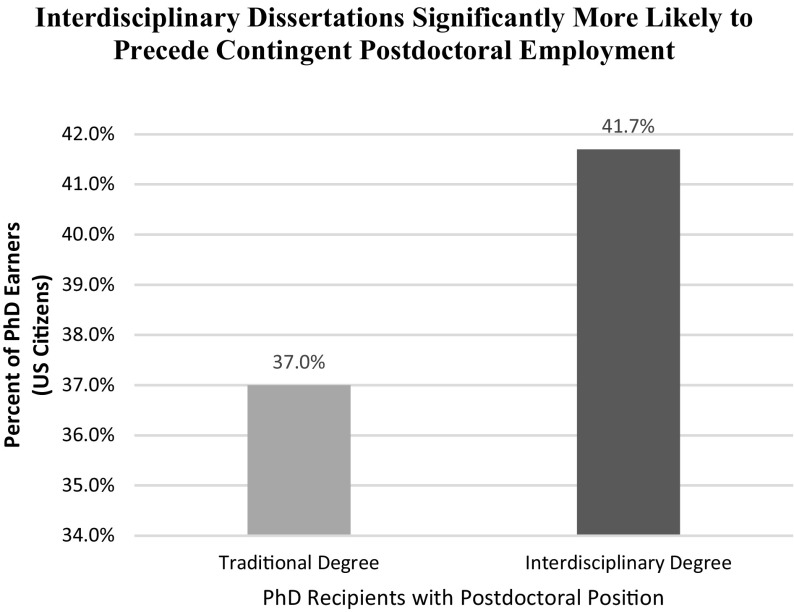
Interdisciplinary dissertations significantly more likely to precede contingent postdoctoral employment

Next, we examine the propensity of individuals in the sample to choose a postdoctoral research position, which postpones full employment for additional training. When we estimate the parameters of Eq. 2 to examine how the various factors affect the decision to pursue postdoctoral work, we find that the probability of an individual accepting a postdoctoral research position after graduation is 4.7% points higher (from 41.7 to 37.0; *p* < 0.001) for those who complete an interdisciplinary dissertation (Table 5; Fig. 3). Similar to our previous analyses, we also find significant effects for the role of gender in this model. Indeed, we find—consistent with previous research (e.g., Moss-Racusin et al. 2012)—that a disproportionate percentage of women and non-white students accept postdoctoral positions.

**Table 5:** Influence of interdisciplinary research upon employment as postdoctoral researcher

Variable	Coefficient	Standard error	*Z*-statistic	*P* value
*Panel A Dependent variable: choose postdoctoral position*				
Interdisciplinary Dissertation	0.15	0.02	8.38	0.00
Father Education	0.01	0.02	0.35	0.72
Mother Education	0.01	0.02	0.68	0.49
Biological Sciences	0.60	0.06	10.29	0.00
Health Sciences	−0.26	0.07	−3.95	0.00
Engineering	−0.27	0.06	−4.50	0.00
Computer Sciences and Mathematics	−0.19	0.06	−2.90	0.00
Physical Sciences	0.38	0.06	6.30	0.00
Social Sciences	−0.19	0.06	−3.36	0.00
Humanities	−0.72	0.06	−12.00	0.00
Education	−0.97	0.06	−15.57	0.00
Business Management	−1.40	0.10	−13.66	0.00
Communications	−1.05	0.10	−10.65	0.00
University w/High Research Activity	−0.19	0.02	−7.77	0.00
University w/Moderate Research Activity	−0.14	0.04	−3.36	0.00
PhD Granting College or University	−0.09	0.04	−2.34	0.02
Birth Year	0.03	0.00	21.14	0.00
Female	0.06	0.02	3.52	0.00
White	−0.18	0.02	−7.98	0.00
Constant	−53.85	2.55	−21.16	0.00
*Panel B Marginal effects of interdisciplinary degree*				
Traditional Degree	37.0%	0.003	117.55	0.00
Interdisciplinary Degree	41.7%	0.005	87.64	0.00

**Fig. 4:**
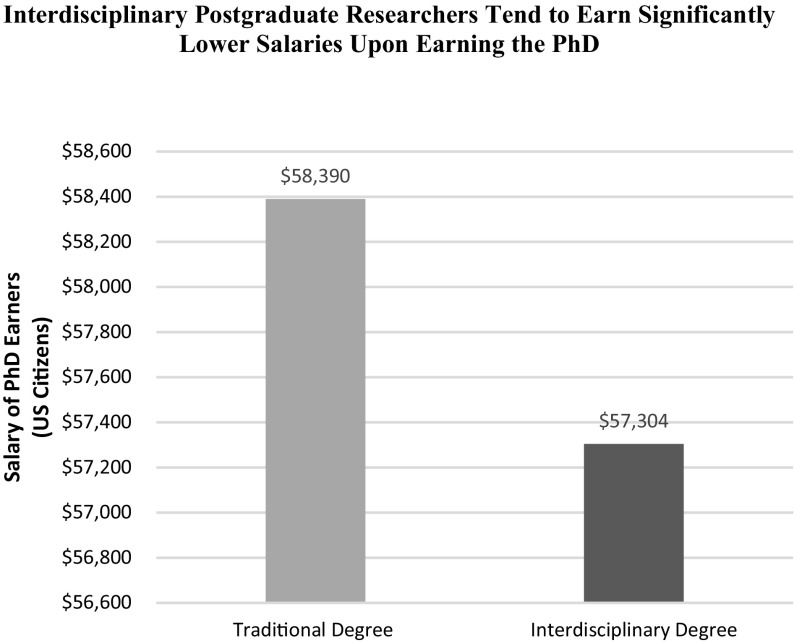
Interdisciplinary postgraduate researchers tend to earn significantly lower salaries upon earning the PhD

Near-term income risk associated with interdisciplinary postgraduate research is indicated by the results in Fig. 4 and supports Hypothesis 1. Individuals who completed risky interdisciplinary dissertation research tend to earn significantly less income in their first year of employment with a doctoral degree. At the margin, individuals who sought an interdisciplinary degree earn an estimated $1086 (approximately 2%) less than those who pursued a traditional degree (from $58,390 to $57,304; *p* < 0.001). Holding research fields and other demographic characteristics constant, Table 6 shows that women tend to earn less compared to men upon completion of the doctorate. Interestingly, European American individuals also earn less in their first year after graduation than those in other racial groups. While there is abundant previous research focused upon the role of gender and ethnicity for salaries among professional employees (e.g., Kulich et al. 2011), our findings for the marginal effects of pursuing interdisciplinary postgraduate research—when controlling for gender and ethnicity—provides novel insight.

**Table 6:** Influence of interdisciplinary research upon salary

Variable	Model 1^a^		Model 2^b^	
	Coefficient	Standard error	Coefficient	Standard error
Interdisciplinary Dissertation	−0.96**	0.33	−0.04	0.30
Postdoctoral Position			22.24***	0.33
Father Education	−0.15	0.37	0.24	0.33
Mother Education	0.25	0.36	0.44	0.32
Biological Sciences	−8.65***	1.16	−1.78	1.04
Health Sciences	8.62***	1.29	6.74***	1.14
Engineering	16.51***	1.20	14.64***	1.07
Computer Sciences and Mathematics	13.34***	1.29	12.19***	1.14
Physical Sciences	1.29	1.20	5.54***	1.07
Social Sciences	0.30	1.15	−1.26	1.02
Humanities	−7.70***	1.19	−13.81***	1.06
Education	7.59***	1.18	1.35	1.05
Business Management	35.02***	1.45	26.48***	1.30
Communications	−0.17	1.64	−7.56***	1.46
University w/High Research Activity	−0.81	0.43	−2.30***	0.38
University w/Moderate Research Activity	3.40***	0.69	1.75**	0.61
PhD Granting College or University	1.12	0.74	0.64	0.66
Birth Year	−0.55***	0.02	−0.32***	0.02
Female	−5.10***	0.32	−4.42***	0.29
White	−1.02*	0.44	−2.16***	0.39
Constant	1139.50***	43.01	648.73***	38.95

* *p* < 0.05; ** *p* < 0.01; *** *p* < 0.001

^a^Results generated from Model 1 represent the first regression in the mediation test

^b^Results generated from Model 2 represent the third regression in the mediation test

Our mediation results indicate that while there appears to be an effect of interdisciplinary research on near-term salaries of new PhD recipients, the choice to accept a postdoctoral position mediates this effect. Our results in Table 6 show that when we include the postdoctoral variable in the salary regression, then the effect of interdisciplinary research disappears. Sobel and Goodman tests confirm that postdoctoral positions mediate the effect of interdisciplinary research (*p* < 0.001).

While income risk is the most objective measure that exists to operationalize Hypothesis 3’s prediction of relatively inferior near-term career outcomes, another outcome to consider is whether or not someone “stays” in academia or goes to industry after earning the PhD. Just as there are numerous reasons why people accept postdoc positions (e.g., Sauermann and Roach 2016), there also certainly exists an array of reasons why people who earn the PhD go to work in industry—indeed, it is significantly more common in some fields than others. To better understand this near-term outcome, Table 3 shows that 7.7% of US citizens in our sample went to work in Industry after earning the PhD and the variable correlates significantly with several others in Table 2—most substantially and positively (*r* = .36, *p* < 0.01) with salary. Beyond those bivariate relationships, we can report that regression analyses that control for disciplinary, demographic, and institutional differences as well as US citizenship show that interdisciplinary dissertators are significantly less likely to go into Industry than others; however, that pattern is sensible in light of the fact that so many interdisciplinary dissertators accept postdoc positions. In Table 6, we can also point out an interesting relationship evident in variables representing the four Carnegie classifications. Individuals who attended a university with moderate research activity reported higher earnings immediately after graduation relative to those who attended universities with extremely high research activity. Results in Table 7 report predicted values for interactions between pursuit of interdisciplinary postgraduate research and the Carnegie classifications. These predicted values were generated from results reported in Tables 3 and 4. Interestingly, a greater proportion of those who attend universities with very high research activity pursued an interdisciplinary PhD and accepted a postdoctoral position, which likely contributes to the lower salary they received.

**Table 7:** PhD recipients from universities with very high research activity were most likely to complete an interdisciplinary PhD

	Received postdoctoral position		Salary	
	Pursuit of interdisciplinary degree		Pursuit of interdisciplinary degree	
Carnegie Classification	No (%)	Yes (%)	No	Yes
Very High Research Activity	38.1	43.0	$58,308	$57,106
High Research Activity	32.7	36.1	$56,957	$57,211
Moderate Research Activity	33.2	39.9	$62,095	$61,029
PhD Granting College or University	34.4	42.1	$60,204	$56,603

## Discussion

The three hypotheses that we examine in this article focus on the question of “who conducts interdisciplinary dissertation research and how do they fare after graduating?” While the Survey data that we used does not include measures that directly identify individuals’ risk preferences or other psychological traits, there is value, both at the institutional and individual level, to better understand the factors that are associated with individuals’ decisions to conduct interdisciplinary academic research. Our hypotheses and related analyses are designed to shed light on such factors across the full population of graduate students in the United States as well as the individuals and institutions that employ and advise them.

### Privileged risk taking

While our results only weakly support privileged risk taking in terms of parental education, it is important to recognize that this variable is only a proxy for parental income and lifetime wealth. In this respect, the significant but weak findings from our study do not negate the evidence from other studies that white males raised by highly educated parents tend to pursue the riskiest degrees (Ball et al. 2010). In the affirmative, the presence of a limited fit with Hypothesis 1 in our analysis of the full population of PhD recipients for a recent year invites closer consideration of the degree to which parental education levels—independent of other important demographic traits—appears to have persistent influence with respect to individuals’ career decisions.

### Entrepreneurial immigrants

In contrast with the relatively limited patterns that were established in relation to Hypothesis 1, our analysis generates findings that are clearly consistent with Hypothesis 2. Graduate students who have immigrated to the United States are disproportionately represented among those who conduct relatively risky interdisciplinary research. Our analysis contributes to understanding the important roles that are played by immigrant populations and offers a valuable complement to debates that often focus on immigrants in relation to manual or “unskilled” labor (e.g., Ndofor and Priem 2011).

### Rewards of risk taking

A common assumption in economic models is that individuals have perfect information, which results in optimal market outcomes; however, ample empirical evidence demonstrates that people often make decisions with imperfect information (e.g., Stiglitz and Weiss 1981). Among entrepreneurs outside academia, Cassar (2010) finds “substantial overoptimism” with respect to the likely chances that an entrepreneur will successfully translate their efforts into a sustainable venture. Among aspiring academics in general, Golde and Dore (2001) find significant mismatches among doctoral students across a wide range of fields when they compared (a) discipline-specific averages for gaining stable, tenure-track employment and (b) individual expectations that a person would gain tenure-track employment. Our results provide a natural extension of Golde and Dore’s (2001) findings and fit with the prediction of Hypothesis 3 whereby we report an apparent gap or mismatch in the near-term rewards that tend to be gained by interdisciplinary researchers upon earning the PhD. Notably, our findings fit with Leahey’s (2007) identification of an inverse relationship between academicians’ salaries and research specialization; however, our analysis builds on previous work by drawing close attention to individuals’ first position after earning the PhD. While our recognition that employment as a postdoctoral researcher mediates the relationship between interdisciplinary dissertating and salary in the year after earning the PhD, the basic fact remains that people who complete an interdisciplinary dissertation fare relatively worse for at least the first year after earning the PhD.

### Limitations and future directions

Limitations of our results that point to directions for future research include our reliance on near-term outcome measures since it is possible that longitudinal studies would demonstrate less unfavorable—and potentially favorable—outcomes for those who completed interdisciplinary dissertations. While the 2010 SED does not permit such analysis, future questions that are worthwhile include: is the distribution of earnings a simple shift in means, or is there greater variance for those pursuing interdisciplinary work? Given that relatively high rewards do clearly accrue to interdisciplinary academic celebrities such as Ariely (2008, 2010) or Diamond (1997) and, more subtly, the senior faculty who demonstrate greater productivity when participating in cross-departmental research centers (Sabharwal and Hu 2013), future research will need to track the pathways of interdisciplinary dissertators past the first year after earning the PhD. For example, while we show a relatively small percentage of graduates are employed in Industry in the first year after earning the PhD, there is ample reason to expect that the percentage is higher several years after earning the PhD given the relatively higher frequency with which people who complete a postdoc position go to work in Industry (e.g., Sauermann and Roach 2016). Given that people with PhDs working in Industry tend to command significantly higher salaries across disciplines (Hanks and Kniffin 2014), it is clear that longitudinal analyses offer value. Looking beyond salary and employment status as outcome variables, future longitudinal research should also examine whether interdisciplinary dissertators might show either better or worse or more divergent patterns of research impact. For example, Wang et al. (2015)—building on Rinia et al. (2001) as well as Tsay and Ma (2003)—show that interdisciplinary research tends to be cited more than other work over longer periods of time (e.g., 13 years post-publication) compared with shorter-terms (e.g., 3 years post-publication). “Sleeping beauty” manuscripts such as those studied by Gorry and Ragouet (2016) similarly help to illustrate the broader trend of lagged success for interdisciplinary research. Future studies that are able to connect such impact-patterns back to whether someone’s postgraduate research was interdisciplinary would shed greater light on this important aspect of academic career paths.

Our focus on one year of data also invites the question of whether cyclical patterns or linear trends might exist with respect to the main findings that we report. For example, just as others have found that members of different ethnic groups variably decide to enter graduate school as a function of business cycles (e.g., Bogan and Wu 2012; Johnson 2013), it is possible that overall economic climates influence the degree to which doctoral students pursue interdisciplinary research. Empirical investigations modeled on our study could investigate whether expansionary economic periods tend to be accompanied by higher-risk interdisciplinary dissertations. Similarly, given the degree to which interdisciplinary research has become more fashionable as a general practice in recent years (e.g., Chen et al. 2014; Huang and Chang 2012; Porter and Rafols 2009; Van Noorden 2015), future research will be useful that examines the impact of this broader linear trend for academic interdisciplinarians.

With respect to the antecedent variables that we were able to consider, our study does not take into account the possibility of pre-existing differences in the intelligence or aptitude of those who conduct interdisciplinary research. For example, while interdisciplinary postgraduate tracks such as the NSF IGERT programs are prestigious and competitive, it is plausible, at least, that students who choose interdisciplinary paths tend to face relatively worse near-term outcomes for reasons that are not due to their interdisciplinary pursuits. A comparison of standardized test scores [e.g., from the Graduate Record Examination (GRE)] that contrasts the populations of those who do and do not complete interdisciplinary postgraduate research would be one way to address this question of omitted variables with respect to potential differences in aptitude. Our analysis—as well as the SED—also omits individual-level variables that measure the performance of specific individuals with respect to any teaching abilities developed in graduate school and any publication record they might have established. While it would be ideal to be able to consider these variables, it is also notable that—in some disciplines, at least—it is typical for graduate students to neither teach nor gain acceptance for a research paper before finishing the PhD (Hamermesh 2013).

Additional trade-offs incurred by our focus on the full sample of earned doctorates rather than individuals from a given discipline include the fact that some combinations of disciplines are more interdisciplinary than others. For example, Vilhena et al. (2014) find that there is greater integration among the social sciences when compared with the ecological sciences. While the justification for our approach is well-illustrated by the fact that there are certainly interdisciplinary researchers who work to integrate “social sciences” and “ecological sciences,” future analyses could check for the robustness of sample-wide patterns by focusing on a subset of broad-scale categories within the full sample. Preliminary analyses that we conducted to examine potential interaction effects across broad disciplinary groups shows that interdisciplinarians in Business appear to be disproportionately penalized compared with those in other disciplinary groupings. This could be a function of the higher market value of traditional PhD-holders in Business relative to other disciplines.

More basically, while our approach for identifying interdisciplinary dissertators follows a field-validated tradition (Falkenheim 2011; Millar 2013; Millar and Dillman 2010a, b), it is possible that the effects that we report in this paper vary as a function of relative interdisciplinarity—or distance between primary and secondary fields (Porter et al. 2007). Consequently, we conducted a separate set of analyses to determine the robustness of our results by operationalizing someone’s dissertation as interdisciplinary only if the secondary field was not within a similar disciplinary grouping and, instead, distantly related from the primary field (e.g., Economics and Evolutionary Biology). When we use this more restrictive definition of interdisciplinary research, we still find that interdisciplinary research affects the decision to pursue postdoctoral research (*p* < 0.001), though the impact on salary is statistically insignificant. Mediation results using this more conservative identification of interdisciplinary research provides outcomes similar to those reported above, thus confirming our original findings that accepting a postdoctoral research position mediates the impact that interdisciplinary research has on salary, though the specific effect that interdisciplinary research has on salary does depend on how such research is classified. While both categorizations of interdisciplinary research have limitations (see Wagner et al. 2011 for more background), this robustness check (1) offers reassurance of the relevance between interdisciplinary research and employment as a postdoctoral researcher and (2) invites future research that utilizes additional methods for classifying research as interdisciplinary. For example, while there are benefits to using self-report data for identifying whether someone’s work is interdisciplinary, it would also be valuable—if feasible—to classify each dissertator’s main product as interdisciplinary (or not) as a function of more objectively available information that is found through their bibliographies (e.g., del Calatrava Moreno et al. 2016; Mugabushaka et al. 2016; Zhang et al. 2015).

Finally, it is important to recognize that our results potentially suffer from selection bias since different disciplines have different incentives for pursuing interdisciplinary research. Thus, the choice to conduct interdisciplinary research is likely a function of the primary field. Unfortunately, the data only include limited information for determining factors that drive the interdisciplinary choice, thus it is likely that unobservable characteristics are also strong determinants. Furthermore, these unobservable traits would remain unobservable in a selection bias adjustment, tainting these estimates as well. Because the user agreement for these data restrict us from merging outside data, it is not possible to rely on another dataset to attempt to capture some of these unobservable characteristics. In addition, this restriction inhibits us from utilizing outside data as instruments to properly identify the decision to pursue interdisciplinary research. We do point out, though, that the fact that the postdoctoral position mediates interdisciplinary research indicates that the salary effect that may be caused by interdisciplinary research is mostly observed through the actual position accepted, and not necessarily the type of research conducted, once disciplinary field is held constant.

## Conclusions

Our analyses provide significant new insights by (1) exploring factors that appear to contribute to the pursuit of interdisciplinary postgraduate research and (2) estimating the near-term consequences for interdisciplinary dissertators. First, we find among US citizens that people with relatively privileged situations, as measured by paternal education levels as well as university prestige, appear more likely to complete an interdisciplinary dissertation. Second, as with people working in industries outside of academia, immigrants appear significantly more likely to be academic risk-takers. With respect to our analyses of antecedent factors, it is also notable that gender is not predictive of decisions to pursue interdisciplinary postgraduate research. In this sense, our findings reject the “women are more holistic” hypothesis that Rhoten and Pfirman (2007) proposed—though we appreciate that it was their primary interest to draw closer systematic attention to the question of individual-level pathways that the current research directly addresses.

With respect to our finding of a salary risk for interdisciplinary dissertators for at least the first year after earning the PhD and our specific finding that the relationship is mediated by interdisciplinarians disproportionately becoming postdoctoral researchers, it is notable that Miller and Feldman (2014) suggest that the relatively low rewards and lack of employment security that accompany the growth in contingent postdoctoral research positions threaten to undermine future development of a scientific workforce. In this regard, while Millar (2013) did not find a greater probability that interdisciplinary dissertators became employed as postdoctoral researchers with her use of data from the 2004 to 2005 and 2006 to 2007 graduating classes, her findings fit the direction and trend whereby our finding of a significant difference among 2010 doctoral recipients seems to reflect a growing tendency for doctorates who complete interdisciplinary dissertations to become contingently employed in the year after earning the PhD.

Beyond contributing to a better understanding of who completes interdisciplinary dissertation research and how they fare after graduating, our findings also have clear relevance for broader policy debates concerning academic career paths. For example, evidence that a greater proportion of immigrants tend to pursue interdisciplinary dissertation research lends itself to endorsements of policies that open more doors for immigrants to doctoral programs in the US. On the other hand, though, evidence for relatively lower salaries—mediated by contingent employment as a postdoctoral researcher—should provide caution (or at least more information) for anyone considering the pursuit or encouragement of interdisciplinary dissertation research. Future research will be needed that examines the longer-term effects of completing an interdisciplinary dissertation; however, the present study provides important baselines for such work.

## Supplementary materials

The command lines that generated the results in this article are available at the CISER Data Archive: https://doi.org/10.6077/J5CISER2779.
